# In Defence of the Normative Account of Ignorance

**DOI:** 10.1007/s10670-022-00529-7

**Published:** 2022-06-26

**Authors:** Anne Meylan

**Affiliations:** University of Zurich, Zurich, Switzerland

## Abstract

The standard view of ignorance is that it consists in the mere lack of knowledge or true belief. Duncan Pritchard has recently argued, against the standard view, that ignorance is the lack of knowledge/true belief that is due to an improper inquiry. I shall call, Pritchard’s alternative account the Normative Account. The purpose of this article is to strengthen the Normative Account by providing an independent vargument supporting it.

## Introduction

The standard view of ignorance is that it consists in the mere lack of knowledge^1^ or true belief.^2^ Duncan Pritchard has recently argued, against the standard view, that ignorance is the lack of knowledge/true belief *that is due to an improper inquiry*. I shall call, Pritchard’s alternative account *the Normative Account*. The purpose of this article is to strengthen the Normative Account by providing an independent argument supporting it.

I shall proceed as follows. In Sect. 2 I present Pritchard’s counterexamples to the Knowledge and True Belief Account and show how these counterexamples lead him to suggest that ignorance also includes a “failing of good inquiry” (Pritchard, 2021: 115). As we shall see, Pritchard’s objection heavily relies on intuitions regarding specific cases and won’t impress those who do not share his intuitions.

Sections 3.1 to 3.3 are the key sections of the paper. There, I present my own argument supporting the Normative Account. This is a two-step argument. First, in Sect. 3.2 I present an objection—the objection of negative value—which shows that some “disvaluable component” needs to be added to the Knowledge/True Belief Account, just as the Normative Account would predict. Second, in Sect. 3.3 I explain why the property of being due to an improper inquiry is such a disvaluable component. In a nutshell, the explanation I favour is as follows. When the absence of knowledge/true belief is due to an improper inquiry, this is a missed opportunity to know/believe something truly. And it is *prima facie* disvaluable to miss the opportunity of achieving something valuable.

As should be clear from this overview, my argument supporting the Normative Account relies on the premise that ignorance is a *prima facie* bad cognitive state or, as Pritchard calls it, an ill. Section 4 is devoted to defending this premise.

## The Knowledge Account, the True Belief Account, and the Normative Account of ignorance

What is ignorance? Two influential views are the following:

*The Knowledge Account*

A subject S is ignorant of the fact that p iff S does not know that p.

*The True Belief Account*

A subject S is ignorant of the fact that p iff S does not hold the true belief that p.

The Knowledge Account and the True Belief Account have distinct strengths. For example, one thing the Knowledge Account has going for it is that it respects common parlance. According to the Oxford English Dictionary (OED), ignorance is the “the fact or condition of being ignorant; want of knowledge (general or special)”. The True Belief Account does not fare so well in this respect. But the True Belief Account is more convincing in other regards. Not all cognitive states that fall short of knowledge intuitively qualify as instances of ignorance. For instance, it does not seem that a subject holding a true justified but Gettierized belief that it is 4 o’clock is ignorant of the time. This is a problem for the Knowledge Account but not for the True Belief Account. According to the True Belief Account, indeed, a true justified and Gettierized belief is not an instance of ignorance.^3^

In a recent paper (2021) Duncan Pritchard presents several counterexamples that target both the Knowledge and the True Belief Account.

The first counterexample is a case in which the subject does not know/believe a pointless truth, such as the number of grains of sand on a beach, even though coming to know/believe this would be possible (with some tenacity). However, it is, according to Pritchard, not clear whether we would ascribe ignorance to the subject in this case. Pritchard asks:

But in passing up knowledge, or even true belief in this proposition would it follow that one is thereby ignorant of this fact? In particular, is there any temptation at all to ascribe ignorance in this case, as opposed to merely noting that the target proposition is something that the agent doesn’t know? Pritchard (2021: 113).

Just like Pritchard, my own intuition is that we would rather simply say that the subject *does not know* how many grains of sand there are and that there is something incongruous in saying that she is *ignorant* of this fact. But this intuition is arguably disputable.

Pritchard offers a second and, in his view, stronger counterexample to the Knowledge/True Belief Account. Some truths cannot be known for structural reasons, no matter how persevering you are. As Prichard points out, some true propositions about the position and momentum of a particle at some specific moments are apparently like this. In such cases, he states, it would be odd to say that we are ignorant of the target proposition.

“given that this doesn’t reflect any cognitive lack on our parts, but rather an epistemic boundary that we cannot cross” Pritchard (2021: 114).

Here again, Pritchard says, we would rather say that we simply *do not know* the position and momentum of the particle.^4^ Here is a similar example that is supposed to drive the same intuition (but more straightforwardly, maybe). It is impossible, for a subject, to know what it felt like to be a newborn.^5^ Now, if someone asks you how it felt, it seems to me that the most natural answer is: “I do not know how it felt when I was a newborn”. Answering “I am ignorant of how it felt when I was a newborn” sounds stilted. And this is so, according to Pritchard, because answering that I am ignorant presupposes that my not knowing is due to some cognitive failure of mine (I say more about Pritchard’s positive suggestion concerning the nature of ignorance just below). And there is no cognitive failure in this example.

In Pritchard’s view, this second type of counterexample also works against the True Belief Account. Even though we could, through sheer luck, come to have a true belief about how it feels to be a newborn, this would not, according to Pritchard, reduce our ignorance. Because, intuitively, we are not ignorant in the first place. We simply do not hold a true belief about this fact.

So, to reiterate, two types of counterexamples contradict the Knowledge and the True Belief Account, according to Pritchard.First, when a subject does not know or does not hold a true belief about a trivial truth, it seems inappropriate to attribute ignorance. Second, there are true propositions that we cannot know but of which we are, intuitively at least, not ignorant.

In the second part of his article, Pritchard makes a positive suggestion as to what ignorance could be, given that it does not merely consist in the absence of knowledge or true belief.

What the cases just considered demonstrate is that there is a normative dimension to ignorance, in the sense that it implies a specific kind of intellectual failing on the subject’s part. In particular, the sort of intellectual failing in question is one concerned with a failing of good inquiry. In all the cases we have looked at, while there is a clear absence of the target epistemic good (whether true belief or knowledge), there is no intellectual failing of the subject qua inquirer in play, and that’s why we don’t attribute ignorance to the subjects concerned. Pritchard (2021: 115).

Let me call this alternative account of ignorance that includes a normative dimension the Normative Account.

*The Normative Account*

Ignorance is the lack of a valuable cognitive state (knowledge/true belief) that is due to an *improper inquiry*.

Here, “improper inquiry” is supposed to mean the same as Pritchard’s lengthier expression “an intellectual failing that is concerned with a failing of good inquiry”. What is an improper inquiry? Answering this question in detail would probably require an article-length treatment. For this paper, the following rough explanation suffices. Inquiry is an activity that has—just like many other activities such as playing certain games and cooking— one (or, perhaps, several) constitutive goal(s). Some philosophers think that the constitutive goal of inquiry is truth; others that it is is knowledge. And there might even be other plausible alternatives such as justification.^6^ I do not need to take a stance in this controversy. Let us suppose here, for the sake of simplicity, that truth is the constitutive goal of inquiry. Saying that truth is the *constitutive* goal of inquiry amounts to saying that some activity of a subject does not qualify as an activity of inquiry if the subject does not aim at getting to the truth of the matter. Since an improper inquiry is an inquiry, an improper inquiry is an activity in which the subject actively pursues the aim of getting to the truth of the matter but in an improper way. When does an attempt to get the truth of a matter qualify as improper? Some commonsensical answers to this question are the following: being too slow while gathering evidence (e.g. laziness, dullness), being careless while gathering evidence, ignoring some evidence (e.g. obliviousness, negligence). In sum, an improper inquiry is one in which the subject S aims at getting to the truth of the matter while treating the evidence at hand in a way that is defective, given S’s purpose.

According to Pritchard, the Normative Account explains our reluctance to attribute ignorance in the cases of pointless truths. When a subject does not know/does not hold a true belief regarding the number of grains of sand on the beach, her not knowing or not holding a true belief is not due to an improper inquiry since she has not inquired and is not even expected to do so. The Normative Account also explains—Pritchard says— our reluctance to attribute ignorance when the subject lacks knowledge of an unknowable truth (e.g. the position and momentum of a particle). When a truth is unknowable, the fact that the subject lacks knowledge is not due to an improper inquiry.

Thus, Pritchard’s view seems to have some explanatory power. However, whether you are impressed by Pritchard’s argument or not clearly depends on whether you share his intuitions regarding his counterexamples. If you do *not* find it unnatural to attribute ignorance in these two kinds of cases, you will not be inclined to embrace the Normative Account. Furthermore, Rik Peels (forthcoming) has recently offered several convincing replies that undermines the effectiveness of Pritchard’s cases.

In the rest of this article, I aim to strengthen the Normative Account by providing an independent argument supporting it. This is a two-step argument. First, in Sect. 3.2, I present an objection—the objection of negative value— which shows that some “disvaluable component” needs to be added to the Knowledge/True Belief Account, just like the Normative Account says. Second, in Sect. 3.3, I explain why the property of being due to an improper inquiry is such a disvaluable component.^7^

## The Normative Account of Ignorance Further Supported

A worry for both the Knowledge and the True Belief Account is that they cannot account for the fact that ignorance is *prima facie* bad or disvaluable (I use these two terms interchangeably). I call this objection “the objection of negative value”. Before presenting this objection, let me clarify what I mean when I claim that ignorance is *prima facie* bad.

### Axiological Clarifications

A *prima facie* bad entity is one that is bad “in virtue of being of a certain kind” (Ross, 2007: 19). The *prima facie* badness of an entity is sometimes defeated. For instance, specific circumstances can turn a *prima facie* bad entity *into an all things considered* good one. Quite uncontroversial examples of *prima facie* bad entities that are sometimes *all things considered* good include the breaking of a promise and painful experiences. There are circumstances in which it is an *all things considered* good thing to break a promise even though breaking a promise is a *prima facie* bad thing to do.

Importantly, then, the claim that ignorance is *prima facie* bad is compatible with the possibility that ignorance is an *all things considered* “bliss” in certain circumstances. For instance, suppose that my friend Edgar used to support the now disbanded left-wing terrorist group RAF. If remaining ignorant of this is the only way to preserve our long-term and rewarding friendship, it might well be, *all things considered*, good to be ignorant of this. Or, to take another kind of example, Peels and Pritchard (2021) have argued that educators should occasionally instil ignorance in their students because this will ultimately enable them to gain more knowledge.^8^ Ignorance might be an *all things considered* good thing in this case too. Just like the breaking of a promise can be *an all things considered* good thing because of its consequences, even though the breaking of a promise remains a *prima facie* bad thing, my ignorance can be an *all things considered* good thing when its *prima facie* final badness is trumped by the goodness of its consequences.

Another clarification concerns the kind of badness that I attribute to ignorance. Is this a form of epistemic badness? The answer to this question does not really matter for my argument below. What I am about to say could be true even if the badness in question is moral. But let me say that I am inclined to answer the above question positively.^9^ If epistemic values have to do with the ideal of intellectual success, of making for ourselves a precise and comprehensive idea of the world around us (which, I take it, is not an absurd view), then there is something epistemically *prima facie* disvaluable in being ignorant.

### The objection of negative value

With these axiological clarifications in mind, let me describe a difficulty encountered by the Knowledge and the True Belief Account alike: they cannot account for the fact that ignorance is a *prima facie* disvaluable cognitive state.

Let us assume, for the sake of the argument, that knowledge and true belief are valuable cognitive states.^10^ This does not automatically make the absence of knowledge/true belief a *prima facie* disvaluable cognitive state. This is because the absence of a valuable entity need not imply the presence of a disvaluable entity.^11^ The absence of a valuable entity might merely imply the presence of a neutral entity. For instance, performing a non-pleasant action does not necessarily mean performing an unpleasant action. Sometimes performing a non-pleasant action means performing an action that is neither pleasant nor unpleasant. Similarly, not knowing or not holding a true belief does not necessarily mean being in a disvaluable cognitive condition. For instance, holding no attitude whatsoever (neither believing, nor disbelieving, nor suspending) regarding a specific proposition p is a way of not knowing that p/not holding the true belief that p. But this is not something disvaluable but rather something neutral. There is nothing even *prima facie* disvaluable in not apprehending a proposition. To think otherwise would be to think that we all hold an incredible number of bad attitudes (think of all the propositions you do not even consider just now).^12^ So even if it is good to know that p and to hold the true belief that p, not to know/not to hold a true belief is not to be in a *prima facie* disvaluable cognitive state. If to be ignorant of a fact is to be in a *prima facie* disvaluable cognitive state (as I assume here and will vindicate later, see Section 4), being ignorant of a fact cannot be the same as not knowing or not holding a true belief. This is a straightforward application of Leibniz’s law.

### Ignorance: The Missed Opportunity to Know

The point made in the previous section is as follows. A suitable account of ignorance needs to explain why ignorance is *prima facie* disvaluable. This is a requirement that neither the Knowledge nor the True Belief Account meets. In contrast, the Normative Account of ignorance can satisfy this desideratum since it does not identify ignorance with the mere absence of knowledge/true belief. To recall, according to the Normative Account, ignorance is the absence of knowledge/true belief *that is due to an improper inquiry*. What still needs to be explained is why this italicized additional property makes the absence of knowledge/true belief disvaluable. One thing is to show that we need an additional component to capture the badness of ignorance. Another is to explain why this additional component makes the whole disvaluable. The purpose of this section is to achieve this second task. I aim to explain why the absence of knowledge/true belief is a bad thing *when it is due to an improper inquiry*.^13^

The explanation I favour is as follows.^14^ When the absence of knowledge/true belief is due to an improper inquiry, this is a missed opportunity. I could have known/held a true belief, but I have missed this chance because I have failed to inquire properly. Now, it is *prima facie* disvaluable to miss the opportunity of achieving something valuable. And this is so even when what you achieve by missing this opportunity would be a neutral achievement if it were not achieved in circumstances that make it a missed opportunity. Let me clarify this last point with an example. As usual, you serve yourselves a glass of water at your university’s canteen. You always drink water with your lunch and neither like, nor dislike, it. When performed in these ordinary circumstances, the action of serving yourself water is *neutral* from the hedonic point of view. It is neither clearly valuable, nor disvaluable (hedonically). During Christmas time, the canteen offers fresh fruit cocktails which is one of your favourite drinks. However, since you do not pay attention, you keep serving yourself water, thereby missing a regular opportunity for gustatory pleasure. What a pity! Because it amounts to a missed opportunity, the action of serving yourself water with your lunch, which in other circumstances is hedonically neutral, is bad in some way.^15^ Furthermore, that there is something *prima facie* disvaluable in missing an opportunity to achieve something valuable is shown by the fact that a missed opportunity is the fitting object of a *negative* emotion, namely, regret. I say more about negative emotions and what they reveal regarding their fitting objects below (Section 4).

Recall that the problematic case for the Knowledge and the True Belief Account is the case in which I hold no attitude whatsoever regarding a specific truth. This is a problematic case because this is a way of not knowing/not holding a true belief that is *prima facie* neutral and cannot, therefore, be identified with ignorance. But to hold no attitude whatsoever regarding a specific truth is to be in a *bad* cognitive state when my being in this condition is due to an improper inquiry. This is because, when my holding no attitude whatsoever regarding a truth is due to an improper inquiry, I could have known instead (had I inquired properly). This is a missed opportunity. So, the Normative Account does not encounter the same difficulty as the Knowledge and the True Belief Account.

### Interlude. The Normative Account and Ignoring

The word “ignorance” and the verb “to ignore” belong to the same lexical family just like e.g. “to know” and “knowledge”, “to construct” and “construction”, etc. Usually, words belonging to the same lexical family denote entities that have some (more or less) tight conceptual connections. For instance, “to construct” refers to a specific action and “construction” denotes the entity that constitutes the achievement of this action (in the sense that the action of constructing X has not been achieved before the construction X exists). Similarly, one can reasonably expect that “ignorance” and “ignoring” denotes entities that are related in some way. In this section, I intend to explore this prospect and show that one noteworthy feature of the Normative Account of ignorance is that it explains the connection between ignorance and ignoring.

The first thing to emphasize is that my ignoring a fact and my ignorance of it are not related in the same way as my knowing a fact and my knowledge of it. While to know that p is one and the same thing as to be in the state of knowledge that p, to ignore the fact that p is not one and the same thing as to be ignorant of the fact that p. To ignore the fact that p (e.g. that your remark hurt your wife, that the rules of chess prohibit this move, etc.) is to refuse to take notice of this fact, to disregard it intentionally (this is the OED’s definition).^16^ And, clearly, to refuse to take notice of something is very different from being simply ignorant of it. Among other differences, the former is a (mental) action while the latter is a state.

Having acknowledged this, one might try to find another connection between ignoring and ignorance by considering whether one is ignorant of the fact that p whenever one ignores the fact that p. But this does not hold either. I can, for instance, ignore the fact that my remarks are hurting my sister without being ignorant of this fact. Remarkably, something opposite seems true. It seems not possible to ignore the fact that p without being aware of this fact. This is because intentionally disregarding something requires that you be aware of it. Let me call this *the awareness condition on ignoring*. The awareness condition on ignoring is acknowledged in very dissimilar contexts. For instance, it grounds this criticism of Lewis’ Rule of Attention^17^:

“One clear defect in the Rule of Attention is that it wrongly equates ignoring something and not being aware of it. If (rudely or quite understandably) I ignore you at a party, this is not because I don’t realise you are there. On the contrary, I have to know you are there to ignore you.” Williams, 1999, 16.^18^

The awareness condition on ignoring is also acknowledged in the literature on self-deception. Self-deceived subjects, while ignoring the evidence supporting the unfavoured truth (the truth that they want to avoid believing), are also aware of this evidence (otherwise self-deceived subjects would not do their best to ignore it). Some authors working on self-deception consider that awareness of the evidence supporting the unfavoured truth is what explains the phenomenological tension that the self-deceived subject typically experiences.^19^ In sum, it seems that one is *not* ignorant of the fact that p when one ignores the fact that p because one is aware of the fact that p, when one ignores the fact that p (and if one is aware of the fact that p, one is not ignorant of this fact).

One interesting feature of the Normative Account of ignorance is that it preserves a connection between ignorance and ignoring (though, I concede, a much looser connection than the two considered in the previous paragraph). As mentioned previously, to ignore the evidence supporting the fact that p is one way of inquiring improperly (among others). Now, when one ignores the evidence supporting the fact that p for a rather long period of time, say t1 to t 25, this tends to lead to a lack of knowledge/true belief regarding p at some future time (t26). If I ignore the evidence supporting the fact that my remarks are hurting my sister for 10 years, I might end up not knowing that I hurt my sister with my remarks. This is, in fact, what happens when self-deception is successful: by ignoring the evidence supporting the truth of p, the self-deceived subject ends up not knowing/not believing that p. Briefly then, the Normative Account of ignorance preserves a connection between ignorance and ignoring in the following way: when my ignoring evidence supporting a fact results in a lack of knowledge/true belief, I am in a state of ignorance, according to the Normative Account.

## Objection: Ignorance is Not *Prima Facie* Bad

Until now, my main purpose was to provide an additional argument supporting the Normative Account. I have proceeded in two steps. First, relying on the objection of negative value, I have tried to show that, to capture the *prima facie* badness of ignorance, a “disvaluable component” needs to be added to the Knowledge/True Belief Account. Second, I have tried to explain why the property of being due an improper inquiry can do this job. In a nutshell, when my not knowing/not holding a true belief is due to my having improperly inquired, this is a bad thing because this is a missed opportunity. I could have known/believed the truth instead.

As should be clear, the whole argument is conditional on accepting the premise that ignorance is a *prima facie* bad cognitive state or, as Pritchard calls it, an ill. In this section I would like to defend this premise.

In his forthcoming book, Rik Peels discusses this premise critically. Peels’ view is that only some kinds of ignorance are *prima facie* bad, not others. Here is what he says:

“Among the varieties of ignorance is what I call disbelieving ignorance: the false belief that p. This is clearly *prima facie* epistemically bad, because falsehood has intrinsic disvalue… However, there are further varieties of ignorance, such as suspending on a true proposition (suspending ignorance), unconsidered ignorance (you fail to truly believe and know that p, simply because you have never considered it)… Of course, these are cases in which one fails to believe the truth and, they therefore lack an important epistemic value. It does not follow…that they thereby have disvalue. And this is exactly what I would say: some cases of ignorance have intrinsic disvalue, whereas others do not”.

Let me first address this criticism. Peels concludes that ignorance is not *prima facie* bad (or that some kinds of ignorance, at least, are not *prima facie* bad) from the view that lacking true belief amounts to being ignorant, that is, from his favourite view of ignorance (The True Belief Account). But it is precisely this view that I deny by relying on the premise that ignorance is *prima facie* bad. It seems, thus, illegitimate to reject the premise that ignorance is *prima facie* bad by relying on the fact that there are non-disvaluable kinds of ignorance according to the True Belief Account. This seems to beg the question against me.

Besides addressing Peels’ criticism, I would like also to give a positive reason to think that ignorance is *prima facie* bad (and this is also true for what Peels calls in the quotation above “unconsidered ignorance”): in a nutshell, ignorance is the fitting object of negative emotions. Before explaining this in detail, it is already worth noting that, in several languages, the literal translation of the English term “ignorance” has a negative connotation. For instance, German distinguishes between “Ignoranz” which denotes a reprehensible and active phenomenon and “Unwissenheit” which denotes the mere absence of knowledge. Similarly, Italian differentiates “ignoranza” which designates a lack of knowledge you *should* have and “nescienza” which rather designates the mere absence of knowledge.^20^

As mentioned previously, the breaking of a promise and painful experiences are rather uncontroversial instances of *prima facie* bad things. One common characteristic of *prima facie* bad things is that they are the *prima facie* fitting objects of negative emotions. Or, to put it differently, it is *prima facie* incorrect to react to a *prima facie* bad thing with a positive emotion. Let me explain. Emotions do not only divide into specific types such as fear, shame, regret, anger, etc. At a more general level, emotions also divide into positive and negative emotions. Joy, admiration and wonder intuitively are positive emotions while regret, anger, shame and fear are negative emotions. Psychologists and philosophers of the emotions often speak of “the *valence* of the emotions”.^21^ Joy, admiration and wonder have a positive valence while regret, anger, and shame have a negative valence. Now, just as each specific emotion is made correct by a specific value property—fear is correct only if it is directed at something dangerous, shame only if it is directed at something shameful, etc.—, at a more general level, a positive emotion is correct only if it is directed at something valuable and a negative emotion is correct only if it is directed at something disvaluable.^22^ This explains, for instance, why it seems *prima facie* inappropriate to feel a positive emotion such as joy, relief or wonder towards the breaking of a promise. Since the breaking of a promise is *prima facie* disvaluable, it is *prima facie* the fitting object of negative emotions. Of course, there are cases in which feeling a positive emotion toward the breaking of a promise seems correct. This happens when the breaking of a promise is considered together with some justificatory circumstances that make it an *all things considered* good thing. But *prima facie* the correct affective reaction toward the breaking of a promise—that is to say, the reaction that is correct when the breaking of a promise is considered “alone”, isolated from its specific circumstances—is to feel a negative emotion. It is only when some specific circumstances make the breaking of the promise useful (for instance) that reacting with a positive emotion might be appropriate.

With this in mind, let us consider what seems to be an uncontroversial case of ignorance.

I am about to take the train. While reaching the platform, I realize that the train has been cancelled. Until this moment I had not even considered this possibility and my having no opinion whatsoever regarding this possibility is due to the fact that I did not check the online schedule. Until this moment, I was ignorant of this fact.

This is a case that qualifies as (unconsidered) ignorance according to the Knowledge Account, the True Belief Account and the Normative Account. Now, let me ask: what is *prima facie* the correct affective reaction in such an uncontroversial instance of ignorance (when the latter is considered, “alone”, isolated from its specific circumstances)? Suppose someone else looks at me on the platform and understands that I was until this moment ignorant of the fact that the train has been cancelled. Does it seem appropriate to react to this with a positive emotion, such as admiration, joy, interest, excitement, hope, or anything one.? It does not seem so. Imagine that this person smiles or manifests relief when she realises that you were ignorant, this seems really inappropriate. Of course, it is possible to amend the example in such a way that it seems appropriate to react with a positive emotion. Just as for the breaking of a promise, it is possible to modify the example in such a way that the *prima facie* badness of my ignorance be trumped by some goodness derived from the specific circumstances of this ignorance. But when we consider the ignorance of someone “alone”, isolated from its circumstances, it does not seem right to feel a positive emotion toward it.

Now, the fact that it seems inappropriate to react to an uncontroversial instance of ignorance with a positive emotion won’t be sufficient to convince those who, like Peels, think that some kinds of ignorance are neutral (that is, neither good nor bad). What I need to show is that it is similarly inappropriate to *feel nothing* in response to my own or another’s ignorance.^23^ Here is thus another question: does it seem appropriate to have no affective reaction at all in response to my own or another’s ignorance? For instance, is it fitting to have no affective reaction when I or someone else realizes that I was ignorant of the fact that the train has been cancelled? Recall that this question should be addressed while isolating the instance of ignorance from the specific circumstances in which it takes place. That is to say, considerations such as the fact that it might make me late to be ignorant of this should be ignored. Suppose then that the cancellation of the train has no effect whatsoever for me because I can simply take the bus instead. Still, is it appropriate to have no affective reaction toward the fact that I was ignorant? Just as it is, for instance, appropriate to have no affective reaction toward the fact that the word “Paris” has 5 letters (a fact that is genuinely *prima facie* neither good nor bad)? I find it quite difficult to accept that my ignorance of the fact that the train has been cancelled deserves the same affective reaction as the fact that the word “Paris” has 5 letters, that is to say, no affective reaction at all. When someone is ignorant of a fact, it does not seem *prima facie* appropriate to simply shrug our shoulders. This, however, is what you need to admit if you take unconsidered ignorance to be a neutral state of mind (neither good, nor bad). On this basis, I conclude that ignorance rather seems to be *prima facie* the fitting object of negative emotions. And this implies that ignorance is *prima facie* disvaluable just as the objection of negative value presented in section 3.2 presupposes.

## Stative vs. Agential Conception of Ignorance

Besides the debate between the Knowledge, the True Belief and the Normative Account, another (even though, as we shall see, closely related) controversy is between the stative and the agential conception of ignorance.^24^ The agential conception of ignorance understands ignorance as *essentially* actively induced, in the sense that it is part of what it is to be an instance of ignorance to be actively brought about. In contrast, the stative conception takes the active inducement to be an *accidental* feature of ignorance (Peels 2018, 15). The stative conception of ignorance is at work in the Knowledge/True Belief Accounts since these accounts takes ignorance to be a state (the state of not-knowing/not-holding a true belief). But the Normative Account is an agential conception. This is because ignorance consists in something that is actively induced (by an improper inquiry), according to the Normative Account. If this is so, the argument supporting the Normative Account (presented in Sects. 3.2 and 3.3) also serves the purpose of bolstering the agential conception.

## Conclusion

The main purpose of this paper was to provide an additional reason to endorse the Normative Account of ignorance. The argument is, in a nutshell, the following: the Normative Account meets a requirement that the Knowledge and True Belief Account do not meet because the Normative Account can explain the *prima facie* badness of ignorance. This is because, when the absence of knowledge/true belief is due to an improper inquiry, I could have known instead had I inquired properly. I have missed the opportunity to know/hold a true belief. And this is a *prima facie* bad thing, as revealed by the fact that it is appropriate to express or feel negative emotions such as regret and disappointment when the opportunity to know/hold a true belief is missed.

## Footnotes

1 See Le Morvan (2011, 2012, 2013), Le Morvan and Peels (2016).

2 See Goldman and Olsson (2009), Peels (2010, 2011, 2012, forthcoming). See Peels forthcoming, chap. 3 for additional references.

3 See Peels forthcoming, chap. 3 for a clear overview of the debate between the Knowledge and the True Belief Account.

4 Similarly, says Pritchard (2021: 114), Wittgensteinian hinge propositions—such as the proposition that I have two hands or that the earth is not going to blow up in two seconds—fall outside, per definition, of the range of propositions for which we can provide reasons and, thereby, out of the range of propositions that we can know. However, we are not ignorant of hinge propositions. We are, in fact, certain of their truth.

5 I assume here that the cognitive limitations of newborns are such that they cannot conceptualise emotions or how things feel even though newborns certainly feel emotions. That is, there is an answer to the question how it feels to be a newborn. But it is impossible to know how they feel because we are not newborns anymore. I do not, for sake of brevity, present a third type of counterexample that Pritchard raises against the Knowledge/True Belief Account. According to Pritchard, we do not know—but are not ignorant of—true propositions that we cannot know for practical reasons. For instance, we do not know—but are not ignorant of—what Caesar had for breakfast before he crossed the Rubicon. See Pritchard (2021: 113).

6 See Mitova (2021) for a fine overview of this debate.

7 I have presented the objection of negative value in more detail elsewhere. See Meylan 2020. The explanation why improper inquiry is a disvaluable component is, however, entirely new, and hopefully more compelling than the explanation sketched in this other paper.

8 In another work, Pritchard (2016) argues that it might be good to be ignorant of certain defeaters when our being conscious of the defeaters in question will hinder knowledge acquisition. If he is right, this is another form of all things considered good ignorance. See also Willard Kyle, unpublished, for the related view that ignorance sometimes contributes to the good epistemic life. Once again, this is not something that I intend to deny. Ignorance can be a *prima facie* disvaluable cognitive state even though it is sometimes an all things considered good thing in virtue of the good consequences that ignorance sometimes has for the conduct of our epistemic life.

9 My prudence is justified by the fact that it is notoriously complicated to explain with any precision what “epistemically” means in statements such as “knowledge, understanding, etc. are epistemically valuable cognitive states” (Cohen 2016). Clarifying this falls outside the scope of this paper.

10 This is a charitable assumption. Both the Knowledge and the True Belief Account would have even more difficulties capturing the badness of ignorance if we did not assume that knowledge and true belief are valuable.

11 For a forceful defence of this point, see Chisholm and Sosa (1966).

12 See Peels forthcoming, chap. 3 for an explicit endorsement of this view.

13 Note that I cannot achieve this task simply by relying on the fact that performing a proper inquiry is a good thing (see Mitova 2021 for an argument supporting this view). As argued in Sect. 3.2., from the fact that performing a certain kind of action is a good thing it does not follow that failing to perform it is bad. Failing to perform it might instead be neutral.

14 I used to explain why a failure to inquire is a *prima facie* bad thing by relying on the epistemic insouciance manifested by such a failure. Peels’s criticism (forthcoming, chap. 3) of this solution leads me to suggest this other, hopefully, more compelling, explanation.

15 That there is a cost in missing an opportunity is an uncontroversial tenet of microeconomics.

16 “To ignore” comes from the Latin verb “ignorare” which means not to know. However, the use of the English verb “to ignore” to express the absence of knowledge is obsolete. By contrast, in French, the verb “ignorer” (which has the same etymology) is still used to denote the state of not knowing. In fact, the French verb “ignorer” can denote both the activity of ignoring something, that is, of deliberately failing to pay attention to something, and the state of not knowing. French seems to be quite isolated in this regard. For instance, the German translation of “to ignore” is “ignorieren” and the latter cannot be used to refer to an absence of knowledge. Apparently, many non-Romance languages function like contemporary German and English. This is, at least, the case of Latvian, Russian, and Hungarian. Thanks to Arturs Logins, Julien Dutant, Sanna Hirvonen, Andras Szigeti for help with the translations.

17 I am grateful to an anonymous reviewer of this journal for this quotation to my attention.

18 See also Levy (2011, 207 and fn. 16) for an additional endorsement of the awareness condition on ignoring.

19 See (Lynch 2012; Funkhouser and Barrett 2016; Noordhof 2009).

20 Polish too distinguishes between “ignorancja”/”ignorowac” which have a negative connotation and “niewiedza” that designates the mere absence of knowledge. Thanks to Johanna de Kalbermatten for her help with the translation.

21 See Colombetti (2005), Deonna and Teroni (2012), de Sousa (1987), Prinz (2010), Tappolet (2000, 2016), Teroni (2011). It is notoriously difficult to explain the intuitive phenomenon of valence. For the purposes of this paper, I can rest content with the rather uncontroversial intuition that some emotions seem positive while others seem negative (I consider the possibility that there are neutral emotions just below).

22 The view that the correctness of an emotion depends on whether it is directed at the appropriate value property is generally attributed to Kenny (1963). Kenny calls the value property that is specific to each emotion “the formal object” of the emotion. In his terminology, dangerousness is the formal object of fear and, therefore, an episode of fear can be correct only if it is directed at something dangerous. See Deonna and Teroni (2012), de Sousa (1987), Mulligan (2007, 2009), Prinz (2004), Tappolet (2000, 2016) for more recent endorsements of this view.

23 I assume here that the appropriate emotional reaction to a neutral state of affairs is to feel no emotion.

24 This issue lies at the heart of a recent discussion between El Kassar (2018) and Peels (2018). For additional distinctions between types of ignorance, see Nottelmann (2016).
